# Supplementary material for: Stacking Model‐Based Classifiers for Dealing With Multiple Sets of Noisy Labels
Source: Biom J. 2025 Mar 12;67(2):e70042. doi: 10.1002/bimj.70042 (PMC11898607; doi:10.1002/bimj.70042)
Supplement: Supplementary file 1 — Supporting Information [file BIMJ-67-e70042-s001.zip › density-based-ensemble-model-main/competitors_real_data/netlab-master/netlab-master/help/errbayes.htm]

Netlab Reference Manual errbayes


# errbayes

## Purpose

Evaluate Bayesian error function for network.

## Synopsis

```
e = errbayes(net, edata)
[e, edata, eprior] = errbayes(net, edata)
```

## Description

`e = errbayes(net, edata)` takes a network data structure
`net` together
the data contribution to the error for a set of inputs and targets.
It returns the regularised error using any zero mean Gaussian priors
on the weights defined in
`net`.

`[e, edata, eprior] = errbayes(net, x, t)` additionally returns the
data and prior components of the error.

## See Also

`glmerr`, `mlperr`, `rbferr`

---

**Pages:**
Index


---

Copyright (c) Ian T Nabney (1996-9)
